# Sesamin, a Naturally Occurring Lignan, Inhibits Ligand-Induced Lipogenesis through Interaction with Liver X Receptor Alpha (LXR*α*) and Pregnane X Receptor (PXR)

**DOI:** 10.1155/2019/9401648

**Published:** 2019-11-25

**Authors:** Tsai-Sung Tai, Ni Tien, Hsin-Yi Shen, Fang-Yi Chu, Charles C. N. Wang, Chieh-Hsiang Lu, Hui-I Yu, Fang-Ping Kung, Hsiang-Hsun Chuang, Ying-Ray Lee, Hsiao-Yun Chang, Yun-Ping Lim

**Affiliations:** ^1^Department of Internal Medicine, Ditmanson Medical Foundation Chia-Yi Christian Hospital, Chiayi 60080, Taiwan, China; ^2^Department of Laboratory Medicine, China Medical University Hospital, Taichung 40458, Taiwan, China; ^3^Department of Medical Laboratory Science and Biotechnology, China Medical University, Taichung 40458, Taiwan, China; ^4^Department of Pharmacy, College of Pharmacy, China Medical University, Taichung 40458, Taiwan, China; ^5^Department of Bioinformatics and Medical Engineering, Asia University, Taichung 40458, Taiwan, China; ^6^Department of Medical Research, Ditmanson Medical Foundation Chia-Yi Christian Hospital, Chiayi 60080, Taiwan, China; ^7^Department of Biotechnology, Asia University, Taichung 40458, Taiwan, China; ^8^Department of Internal Medicine, China Medical University Hospital, Taichung 40458, Taiwan, China; ^9^Department of Medical Research, China Medical University Hospital, Taichung 40458, Taiwan, China

## Abstract

Liver X receptor (LXR) is a nuclear receptor that regulates various biological processes, including de novo lipogenesis, cholesterol metabolism, and inflammation. Selective inhibition of LXR may aid the treatment of nonalcoholic fatty liver disease (NAFLD). Sesamin is a naturally occurring lignan in many dietary plants and has a wide range of beneficial effects on metabolism. The mechanism underlying sesamin action especially on the regulation of LXR remains elusive. Reporter assays, mRNA and protein expression, and in silico modeling were used to identify sesamin as an antagonist of LXR*α*. Sesamin was applied to the hepatic HepaRG and intestinal LS174T cells and showed that it markedly ameliorated lipid accumulation in the HepaRG cells, by reducing LXR*α* transactivation, inhibiting the expression of downstream target genes. This effect was associated with the stimulation of AMP-activated protein kinase (AMPK) signaling pathway, followed by decreased T0901317-LXR*α*-induced expression of SREBP-1c and its downstream target genes. Mechanistically, sesamin reduced the recruitment of SRC-1 but enhanced that of SMILE to the SREBP-1c promoter region under T0901317 treatment. It regulated the transcriptional control exerted by LXR*α* by influencing its interaction with coregulators and thus decreased mRNA and protein levels of genes downstream of LXR*α* and reduced lipid accumulation in hepatic cells. Additionally, sesamin reduced valproate- and rifampin-induced LXR*α* and pregnane X receptor (PXR) transactivation. This was associated with reduced expression of target genes and decreased lipid accumulation. Thus, sesamin is an antagonist of LXR*α* and PXR and suggests that it may alleviate drug-induced lipogenesis via the suppression of LXR*α* and PXR signaling.

## 1. Introduction

The liver plays a major role in the systemic lipid homeostasis by regulating lipogenesis, lipolysis, and fatty acid oxidation. The disruption of these processes may lead to pathological conditions. For example, an abnormal accumulation of lipids in the liver in people who drink little or no alcohol characterizes nonalcoholic fatty liver disease (NAFLD) [1, 2]. Hepatocytes affected by this disease show a pathological accumulation of vacuoles filled with triglycerides (TGs). Such excessive lipid stores are strongly associated with the observation of clinically abnormal livers assessed by hepatic function tests. The incidences of NAFLD have been increasing globally with a prevalence of about 25–45% in the normal adult population, and this trend accompanies the prevalence of obesity, type 2 diabetes, and cardiometabolic abnormalities [1]. More than 10% of NAFLD cases may progress to nonalcoholic steatohepatitis (NASH), which may eventually lead to liver cirrhosis and hepatocellular carcinoma [1]. Chronic exposure to steatogenic drugs impairs the hepatic regeneration capacity and potentially leads to the development of NAFLD [3]. Indeed, a drug-induced fatty liver is one of the common forms of liver injury observed clinically. From a molecular point of view, several genes involved in the disrupted fatty acid synthesis associated with NAFLD, such as sterol regulatory element-binding protein 1c (SREBP-1c), and a series of downstream target genes, such as stearoyl-CoA desaturase-1 (SCD), acetyl-CoA carboxylase (ACC), and fatty acid synthase (FAS), are upregulated in NAFLD patients [4, 5].

Lipid synthesis and oxidation in the liver are finely regulated by various enzymes and transcription factors to achieve homeostasis. The dysregulation of lipid metabolism via interactions of drugs with key regulators of lipid homeostasis, including members of the nuclear receptor (NR) family, such as liver X receptors (LXR) and pregnane X receptor (PXR), has been reported [6]. The liver X receptors (including LXR*α*, *NR1H3*, and LXR*β*, *NR1H2*) are members of the NRs superfamily [7]. Their expression patterns differ, and LXR*β* is expressed ubiquitously, whereas LXR*α* expression is restricted to metabolic-related tissues, including liver, intestine, kidney, and adipose tissue. The main roles of LXRs are the regulation of cholesterol efflux and transport, as well as control of hepatic lipogenesis. Compared with LXR*β*, LXR*α* acts as a major sensor for lipid homeostasis, as observed in an LXR*α* null mice model [7]. Furthermore, LXR*α* induces the expression of SREBP-1c [7]. SREBPs form a family of membrane-bound, basic helix-loop-helix leucine zipper transcription factors. In the liver, SREBP-1c is abundantly expressed, where it plays a major role in fatty acid synthesis. Moreover, SREBP-1c regulates the expression of various lipogenic genes, including ACC, FAS, SCD, ATP citrate lyase (ACLY), and fatty acid elongase (FAE) [7]. Thus, these LXR*α* and SREBP-1c are closely related to the regulation of lipid metabolism.

During their activation, LXRs heterodimerize with the retinoid X receptor (RXR, *NR2B1*), translocate into nucleus, bind to a LXR response element, which consists of two hexameric nucleotide direct repeats separated by four nucleotides (DR4), and then promote the transcription of target genes [7]. Oxysterols are the main physiological activators of LXRs. Researchers have explored this pathway to develop synthetic LXR agonists, which have been shown to present antiatherosclerotic effects. Such effects are explained by the ability of these agonists to control the expression of components of the reverse cholesterol transport system (RCT) [8]. This transport system depends on the ATP-binding cassette transporter A1 (ABCA1) or G1 (ABCG1), which is involved in cholesterol efflux from nonhepatic peripheral tissues and macrophages to the liver through the formation of mature high-density lipoproteins (HDL). Induction of intestinal RCT-related genes expression upon LXR*α* activation reduces the efficiency of cholesterol absorption and hence promotes fecal cholesterol disposal [8]. However, activation of LXR by these synthetic ligands in mice leads to undesired side effects, such as hepatic lipogenesis and hypertriglyceridemia, due to increased expression of lipogenic genes, such as FAS and SREBP-1c. Indeed, mice treated with synthetic LXR agonists, e.g., T0901317 (T090), showed increased TGs levels via upregulation of these genes [9]. Although the effect on plasma TGs was transient, that on hepatic TGs was persistent and led to severe liver steatosis and dysfunction. Thus, the development of LXR agonists as antiatherogenic agents warrants further research.

The activity of LXRs is further affected by another layer of control, the interaction with corepressors and coactivators. The LXR/RXR heterodimer is normally bound to corepressors or coactivators in the absence or presence of agonists, respectively [10]. Unliganded LXR associated with corepressors, including nuclear receptor corepressor (NCoR)/silencing mediator of retinoic acid and thyroid receptor (SMRT), recruits histone deacetylase and thus represses gene transcription. Upon ligand binding, the corepressors are dissociated, and coactivators, such as steroid receptor coactivator-1 (SRC-1), are recruited. This relieves chromatin-mediated repression and initiates gene transcription. Corepressors and coactivators might have significant effects on the activity of LXRs. For example, small heterodimer partner-interacting leucine zipper protein (SMILE), a novel corepressor of LXR*α*, can be induced through the hepatic AMP-activated protein kinase (AMPK) pathway upon ursodeoxycholic acid treatment and can inhibit the expression of hepatic lipogenic genes [11]. The “metabolic master switch,” AMPK, is a phylogenetically conserved serine/threonine protein kinase that mediates cellular adaptation to environmental and nutritional stress [12]. It is a heterotrimeric complex composed of a catalytic *α* subunit and two regulatory subunits *β* and *γ*. A large variety of this heterotrimeric combination arises from each combination of subunit isoforms (*α*1, *α*2, *β*1, *β*2, *γ*1, *γ*2, and *γ*3). Phosphorylation of threonine 172 (Thr172) by upstream kinases (LKB1, CAMKK*β*, and others) activates AMPK and results in the inhibition of hepatic fatty acid synthesis [13]. One of the main mechanisms underlying this effect is the AMPK-mediated suppression of SREBP-1c and reduced expression of downstream lipogenic gene. Therefore, lipid metabolism homeostasis is regulated at both transcriptional and posttranslational levels, and this regulation is mediated by several molecular players, such as NRs, ligands, coactivators, corepressors, and kinases.

Sesamin (SSM) is a major fat soluble furofuran-type lignan abundantly found in sesame seeds (*Sesamum indicum* L.) and derived products (e.g., sesame seed oil) [14]. This molecule is also present in several plants distributed in different genera, including, Camellia, Magnolia, Piper, Sesamum, and Virola [15]. The beneficial effects of sesamin have been reviewed by several researchers [14]. Compelling *in vitro* and *in vivo* experimental evidence indicates that sesamin has significant antioxidant, anti-inflammatory, hypoglycemic, antihypertensive, antiestrogenic, proapoptotic, and proautophagic activities. Moreover, it presents anticancer properties against multiple cancer types. In addition to these effects, sesamin has also been found to affect lipid metabolism, including the amelioration of blood lipid profile and the improvement of the recovery from liver steatosis. Furthermore, sesamin have been shown to present antiatherogenic effects due to its modulation of macrophage cholesterol homeostasis.

The molecular pathways involved and the outcome of such an LXR*α* antagonist remain unexplored. Therefore, to assess the suitability of sesamin to target the LXR*α* pathway and its downstream effects, we evaluated the effect of sesamin on the transactivation of LXR*α* and on the mRNA and protein expression of lipogenic genes. We used two cell lines (hepatocytes and intestinal epithelia) to allow the identification of potentially different effects on hepatic and intestinal tissues. Moreover, *in silico* modeling was used to predict the interaction between sesamin and LXR*α* and the potentiality of used upon drug-induced lipogenesis were also been evaluated.

## 2. Materials and Methods

### 2.1. Chemicals and Cell Culture

All chemicals used in this study were of the highest purity grade available from Sigma-Aldrich (St. Louis, MO, USA). Sesamin (SSM, with purity ≥95%), T0901317 (T090, with purity ≥98%), valproate (VPA, sodium salt, with purity ≥98%), and rifampin (RIF, with purity ≥97%) were dissolved in dimethyl sulfoxide (DMSO) or ultrapure water at appropriate concentrations as stock solutions just before use. Human hepatocellular carcinoma cell line, HepG2, and human intestinal cell line, LS174T, were obtained from the Food Industry Research and Development Institute (FIRDI, Taipei, Taiwan, R.O.C) and Cell Lines Service (Cell Lines Service GmbH, Eppelheim, Germany), respectively. These two cell lines were cultured in the alpha-minimum essential medium (*α*MEM; Gibco BRL) supplemented with 10% (v/v) fetal bovine serum (FBS; Gibco BRL, Grand Island, NY) and 1 × L-GlutaMAX™ (Thermo Fisher Scientific, Waltham, MA) freshly added before use and without antibiotics. A single use hepatoma cell line, HepaRG™ cells, was obtained from Thermo Fisher Scientific (Waltham, MA, USA). Once received, the cells in the frozen vials were thawed and seeded into a culture dish (10 cm) and maintained in William's E medium (Sigma-Aldrich, St. Louis, Missouri, USA), supplemented with freshly added 10% (v/v) FetalClone^II^ serum (Hyclone™, GE Healthcare, Chicago, Illinois), 50 *μ*M hydrocortisone hydrogen succinate, 5 *μ*g/mL human insulin, and 1 × L-GlutaMAX (all of them are freshly added) for about 2 weeks until confluency was achieved. In the following 2 weeks, cells were continuously maintained in the above-mentioned medium with 2% (v/v) DMSO to induce differentiation to hepatocyte-like properties. Cells were incubated under an atmosphere of 5% CO_2_ at 37°C. Cell viability was assessed using para-nitrophenylphosphate (PNPP) as previously reported [16].

### 2.2. Transient Transfection and Reporter Gene Assay

HepG2 cells were transfected with the following plasmids, 0.04 *μ*g human full-length LXR*α* [16] or human PXR [17] expression vector, with 0.15 *μ*g of promoter constructs, including 3 × LXRE-*tk*-Luc [7], SREBP-1c-Luc [18], S14-Luc [19], or SCD-Luc [20]. All transfections were cotransfected with 0.02 *μ*g of internal reporter plasmids for normalizing transfection efficiencies based on *β*-galactosidase (*β*-gal) gene by using PolyJET™ transfection reagent (SignaGen Laboratories, Rockville, MD) as recommended by the manufacturer. In brief, cells were exposed to drug treatments after 6-7 h since transfection, and the exposure was maintained for 24 h. Cell lysates were analyzed for luciferase activity using the luciferase assay detection kit (Promega, Madison, Wisconsin, USA) according to manufacturer's instructions.

### 2.3. Oil Red O Staining

To assess the intracellular neutral lipid content, we treated differentiated HepaRG cells with various combinations of drugs according to the protocol shown in [Supplementary-material supplementary-material-1]. Briefly, confluent cells exposed to 2% (v/v) DMSO for 2 weeks were then treated with T090 (10 *μ*M), VPA (693.4 *μ*M), or RIF (20 *μ*M) alone or in combination with 5 or 10 *μ*M SSM for additional 14 days. Oil red O staining was performed as described previously [17]. In brief, after the end of treatment, cells were fixed with 10% (v/v) formalin and rinsed with phosphate-buffered saline (PBS). After completely dried, the cells on slides were stained with Oil Red O (diluted in 60%, v/v, isopropanol) for 20 min at 20°C to 25°C in the dark. After another drying step, images were acquired under a phase-contrast microscope (Axio Observer A1, ZEISS, Inc. Germany) at 400x magnification. Then, lipids were extracted using 100% isopropanol, transferred to microplates, and read at 510 nm using a multimode spectrophotometer.

### 2.4. RNA Extraction and Quantitative Real-Time PCR (qRT-PCR)

For the determination of gene expression levels, total RNA was extracted using a Direct-zol™ RNA MiniPrep kit (Zymo Research, Irvine, CA, USA) according to the protocol provided by the manufacturer. Total RNA was reversed transcribed using a High Capacity cDNA Reverse Transcription kit (Applied Biosystems™, Thermo Fisher, Waltham, MA, USA). The resulting cDNA was amplified by qRT-PCR using Luminaris Color HiGreen qPCR master mix (ThermoFisher Scientific, Waltham, MA) in a StepOnePlus™ Real-Time PCR System (ThermoFisher, Waltham, MA, USA) using standard procedures.  Table 1 shows the sequences of each primer used in this study. The expression levels of target genes were normalized by *β-actin* mRNA levels.

### 2.5. Western Blotting

Western blot was performed as described previously [17] using samples from differentiated HepaRG cells treated with T090 alone or in combination with SSM for 24 h. Total protein samples were obtained after RIPA buffer extraction. The buffer was supplemented with a phosphatase inhibitor cocktail for phosphorylated protein detection. Then, samples were subjected to electrophoresis in 10% SDS-PAGE gels. The separated proteins were then transferred to a nitrocellulose membrane (Amersham Biosciences, Buckinghamshire, UK). Membranes were incubated with the following primary antibodies: anti-SREBP-1c, ACLY, FAE, ACC, and S14 antibodies (purchased from Novus Biologicals, Centennial, CO, USA), FAS, SCD, SRC-1, and *β*-actin antibodies (purchased from Santa Cruz, Dallas, TX, USA), and SMILE and phosphoAMPK antibodies (purchased from GeneTex, Irvine, CA, USA). Then, the blots were probed with the appropriate secondary antibodies, and bands were visualized using an enhanced chemiluminescence reagent (Merck Millipore, Billerica, MA).

### 2.6. Nuclear Extract Preparation and DNA Affinity Precipitation Assay (DAPA)

Treated cells were washed twice with ice-cold PBS and collected in 500 *μ*L PBS. The cells were pelleted by centrifugation at 7,500 ×*g* for 20–30 s, resuspended in 400 *μ*L of buffer A (10 mM HEPES (pH 7.9), 1.5 mM MgCl_2_, 10 mM KCl, and with 1 × protease inhibitor cocktail), and left on ice for 10 min. The nuclei were pelleted by centrifugation at 7,500 ×*g* for 20–30 s, resuspended in 100 *μ*L of buffer C (20 mM HEPES (pH 7.9), 1.5 mM MgCl_2_, 0.2 mM EDTA, 420 mM NaCl, and 25% (v/v) glycerol, with 1 × protease inhibitor cocktail), and incubated on ice for 20 min. The nuclear extracts were obtained by centrifugation at 7,500 ×*g* for 30 min and stored at −80°C until analysis. We used DAPA to identify the interaction between transcription factors and coregulators and to assess binding to the specific promoter response elements and the effects of drug treatments on binding. Biotinylated oligonucleotides covering the LXR*α SREBP-1c* LXRE oligonucleotides (for HepaRG cells, 5′-biotin-CAG TGA CCG CCA GTA ACC CCA GC-3′) or *ABCG1* LXRE oligonucleotides (for LS174T cells, 5′-biotin- GGC AAG AGG TAA CTG TCG GTC AAA TCC T-3′) were synthesized and used as probes. Nuclear extracts (500 *μ*g) from treated HepaRG and LS174T cells were exposed to T090 (10 *μ*M) alone or in combination with 20 or 40 *μ*M SSM for 24 h and incubated with these biotinylated probes (2 *μ*g each) and 20 *μ*L of 50% (w/v) streptavidin-agarose beads slurry (Sigma-Aldrich, St. Louis, MO, USA). The mixture was incubated at room temperature for 1 h at 6 rpm. Then, the beads were pelleted and rinsed with ice-cold PBS three times. Bound protein complexes were eluted using 1 × SDS-PAGE sampling buffer and then analyzed by Western blotting analysis with specific antibodies against SRC-1 and SMILE.

### 2.7. Molecular Docking

Computational methods allow the identification of potentially novel NRs ligands. We investigated the steric and electrostatic complementarity between the ligand-binding domain of LXR*α* and putative ligands using molecular docking. The analysis was based on the existing structure of heterodimeric complex of LXR*α* and RXR*β* (PDB entry 1UHL), which was used as a template to docking of SSM and putative ligands using Discovery Studio 4.5. After removing all crystallized H_2_O molecules from the former construction, hydrogen was added into the CDOCKER module. This module is a powerful CHARMm-based docking method that has been used to generate highly accurate docking poses. In this refinement application, the ligands were conceded to tilt around the rigid receptor [21].

### 2.8. Statistical Analysis

Results are presented as mean ±standard error (SE). Multiple comparisons were performed using one-way analysis of variance (ANOVA) followed by Fisher's restricted least significant difference (LSD) procedure. For all analyses, *p* values below 0.05 were considered to indicate statistical significance. Analyses were performed using SPSS software for Windows (SPSS version 20.0, Armonk, NY, USA).

## 3. Results

### 3.1. Sesamin Decreased T0901317-Induced Lipid Accumulation in Differentiated HepaRG Cells and Attenuated LXR*α* Transactivation

**Figure 1:**
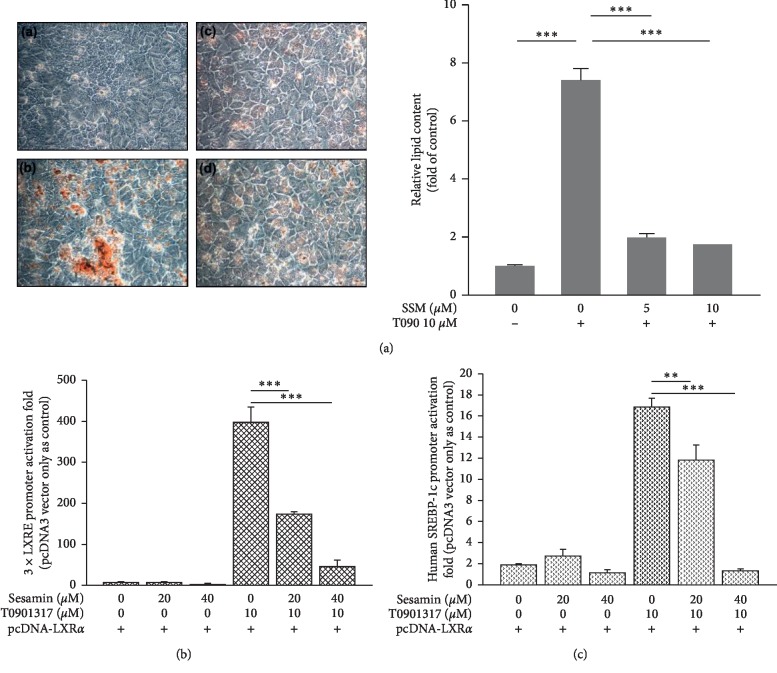
Lipid accumulation in differentiated HepaRG cells and transcriptional assays of 3 × LXRE and SREBP-1c reporter activity in HepG2 cells to determine the effects of sesamin (SSM) and T0901317 (T090)-mediated activation of LXR*α*. (a) Differentiated HepaRG cells were repeatedly exposed to (A) solvent, (B) 10 *μ*M T090, (C) 10 *μ*M T090 + 5 *μ*M SSM, and (D) 10 *μ*M T090 + 10 *μ*M SSM for 14 days. Lipid accumulation was then assessed using Oil Red O staining, which allows the detection of triglycerides and cholesterol esters. HepaRG cells were observed and photographed under a phase‐contrast microscope (original magnification ×400). Oil red O dye was then extracted using isopropanol and quantified with a microplate reader at 510 nm. Values represent the mean ± SE; *n* = 3; ^*∗∗∗*^*p* < 0.001 compared with control or T090-treated groups as indicated. HepG2 cells were cotransfected with a LXR*α* expression plasmid and (b) 3 × LXRE-Luc and (c) SREBP-1c-Luc reporter genes and treated with 20 or 40 *μ*M SSM alone or in combination with 10 *μ*M T090 for 24 h. Data represent the mean ± SE; *n* = 4; ^*∗∗*^*p* < 0.01; ^*∗∗∗*^*p* < 0.001 compared with control or T090-treated groups as indicated.

In a previous work, SSM was found to be cytotoxic to certain cancer cells [22]. Thus, we first performed acid phosphatase assays to evaluate the possible cytotoxicity of SSM against the cell lines used in this study (HepG2, HepaRG, and LS174T). In cells incubated with SSM (10–40 *μ*M) alone or in combination with 10 *μ*M T090 or 20 *μ*M RIF for 24 h, cell viability remained above 80% (Figures [Supplementary-material supplementary-material-1]–[Supplementary-material supplementary-material-1], respectively) as compared with control untreated cells. However, 20 and 40 *μ*M SSM had cytotoxic effects on cells during long-term treatment (data not shown). Thus, we proceeded to use low doses (5 and 10 *μ*M) of SSM for long-term treatments. Then, cells were continuously exposed to treatments for 14 days, and Oil Red O staining was used to detect intracytoplasmic lipid droplets. In order to assess the lipid content in HepaRG cells, we treated the cells with 10 *μ*M T090, a synthetic LXR*α* agonist, alone or in combination with 5 or 10 *μ*M SSM. As shown in Figure 1(a), T090 significantly induced the accumulation of neutral lipid by 7.4-fold compared with the control cells. However, this effect was attenuated by 74% and 77% in cells cotreated with 5 and 10 *μ*M of SSM, respectively.

Next, we performed LXR*α* reporter assays to confirm the effects of SSM on LXR*α* transactivation. Human construct containing three repeats of the minimal direct repeat 4 (DR4) motif (3 × LXRE-*tk*-Luc) and human SREBP-1c promoter that harbors LXR*α* binding sites were cotransfected with full-length huma LXR*α* expression plasmids into HepG2 cells. We investigated the inhibitory effects of SSM on LXR*α* transactivation-related 3 × LXRE-*tk*-Luc and SREBP-1c-*tk*-Luc promoter activity in the presence of LXR*α*, T090, and SSM. We observed a significant transactivation of both 3 × LXRE-*tk*-Luc and SREBP-1c-*tk*-Luc promoter activity (396.4-fold and 16.8-fold, respectively) in cells treated with T090 (Figures 1(b) and 1(c)). However, these enhancements were attenuated by the addition of SSM in a dose-dependent manner (Figures 1(b) and 1(c)). These results indicate that SSM treatment reduced the cellular lipid content in hepatocyte-like cells through inhibition of LXR*α*-transactivation.

### 3.2. Sesamin Decreased T0901317-Induced Hepatic Lipogenic Gene Expression at mRNA and Protein Levels

**Figure 2:**
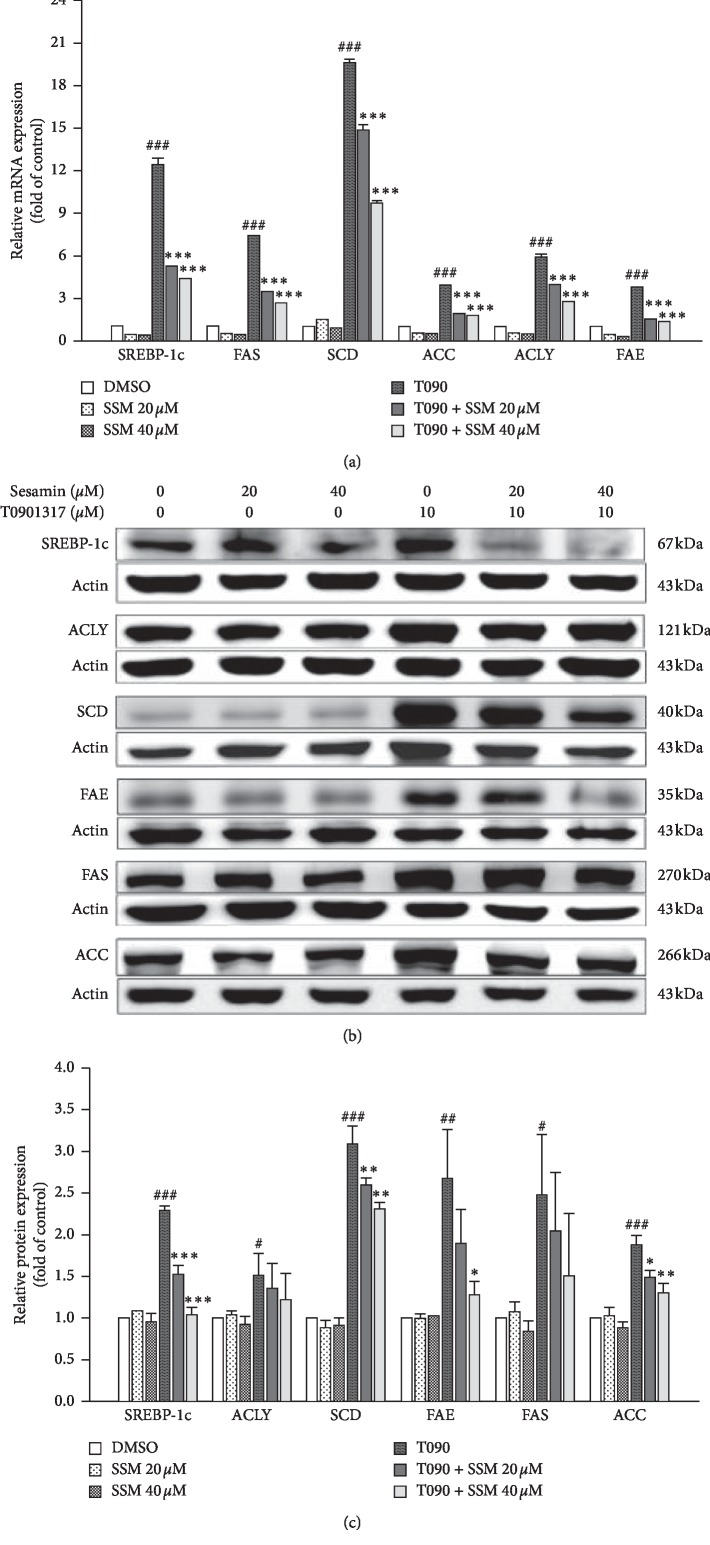
Sesamin (SSM) inhibits the mRNA and protein expression of T0901317 (T090)-induced LXR*α* downstream genes. Differentiated HepaRG cells treated with 20 or 40 *μ*M SSM alone or in combination with 10 *μ*M T090 for 24 h. (a) Quantitative real-time PCR results of gene expression levels of *SREBP‐1c*, *FAS*, *SCD*, *ACC*, *ACLY*, and *FAE*. *β-actin* was used as an internal control. Data represent the mean ± SE; *n* = 3; ^###^*p* < 0.001; ^*∗∗∗*^*p* < 0.001 compared with control or T090-treated groups, respectively, as indicated. (b) and (c) Differentiated HepaRG cells treated with SSM and T090, either individually or in combination for 24 h, were harvested, and their protein expression levels were analyzed via western blot. Quantitation of the indicated protein bands was corrected by *β*-actin expression. The representative blot shown was quantified with ImageJ software. Data represent the mean ± SE; *n* = 3; ^*∗*/#^*p* < 0.05; ^###/*∗∗∗*^*p* < 0.001 compared with control or T090-treated groups, respectively, as indicated.

From the reporter assay results, we confirmed that SSM inhibits LXR*α* activity. To test whether this inhibitory effect also affects the LXR*α*-mediated lipogenic target genes expression, differentiated HepaRG cells were treated with T090 alone or in combination with SSM for 24 h. Quantitative real-time PCR assays were used to measure the mRNA levels of *SREBP-1c* and a set of target genes, including *FAS*, *SCD*, *ACC*, *ACLY*, and *FAE*. As shown in Figure 2(a), T090 treatment significantly induced the expression of those genes by 12.5, 7.4, 19.6, 3.9, 5.9, and 3.7 folds, respectively, compared with control cells. However, the upregulation of these genes was attenuated in the presence of SSM (Figure 2(b)). We further assessed protein levels of SREBP-1c, ACLY, SCD, FAE, FAS, and ACC to investigate the inhibitory effects of SSM in differentiated HepaRG cells. Similar to qRT-PCR results, T090 treatment significantly induced the protein levels of these target genes (Figures 2(b) and 2(c)). Moreover, SSM decreased levels of these proteins in a dose-dependent manner, which is consistent with its effect at the transcriptional level (Figures 2(b) and 2(c)). These findings suggest that SSM inhibited the LXR*α*-SREBP-1c signaling pathway and prevented the expression of a set of related genes at both mRNA and protein levels. Since LXR*α* inhibition may affect the RCT, which is associated with hypercholesterolemia, we assessed the effect of SSM exposure of T090-treated HepaRG cells. We found that SSM did not significantly affect the expression levels of ABCG1 gene expression in these cells ([Supplementary-material supplementary-material-1]).

### 3.3. Molecular Interactions between LXR*α* and Sesamin

**Figure 3:**
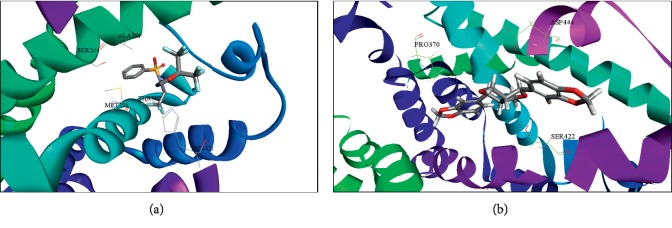
Molecular docking analysis of T0901317 (T090) and sesamin (SSM) with LXR*α*. Superimposition of docking posed of compounds in the LXR*α*-binding pocket of 3D structure (PDB entry 1UHL). (a) Pose 3D structure of agonist T090 in the active site of LXR*α* ligand-binding domain. (b) Docking pose of SSM in the active site of LXR*α* ligand-binding domain.

Two ligands, T090 and SSM, were virtually docked to the crystal structures of ligand-binding domain (LBD) of LXR*α* (PDB entry 1UHL) using the docking program CDOCKER separately. The docking results showed that T090 (Figure 3(a)) and SSM (Figure 3(b)) binding to the active site of LXR*α* had CDOCKER energy scores of 45.7082 (T090) and 33.2242 (SSM), and binding energies were 19.8764 (T090) and 19.2461 (SSM) Kcal/mol. The binding model clearly indicated that the interaction of T090 with the LBD involved residues such as SER263, ALA261, MET298, THR302, and HIS421. On the other hand, SSM binding to the LBD depends on PRO370, ASP444, and SER422 residues. Therefore, T090 and SSM possess distinct predictive binding patterns for the LXR ligand binding domain and might recruit different coregulators. The molecular docking analysis demonstrated that SSM could act as a novel LXR*α* ligand, providing insight for the design of novel LXR*α* modulators.

### 3.4. Sesamin Increased the Expression of Reverse Cholesterol Transport-Related Genes (ABCA1 and ABCG1) in Intestinal Cells

**Figure 4:**
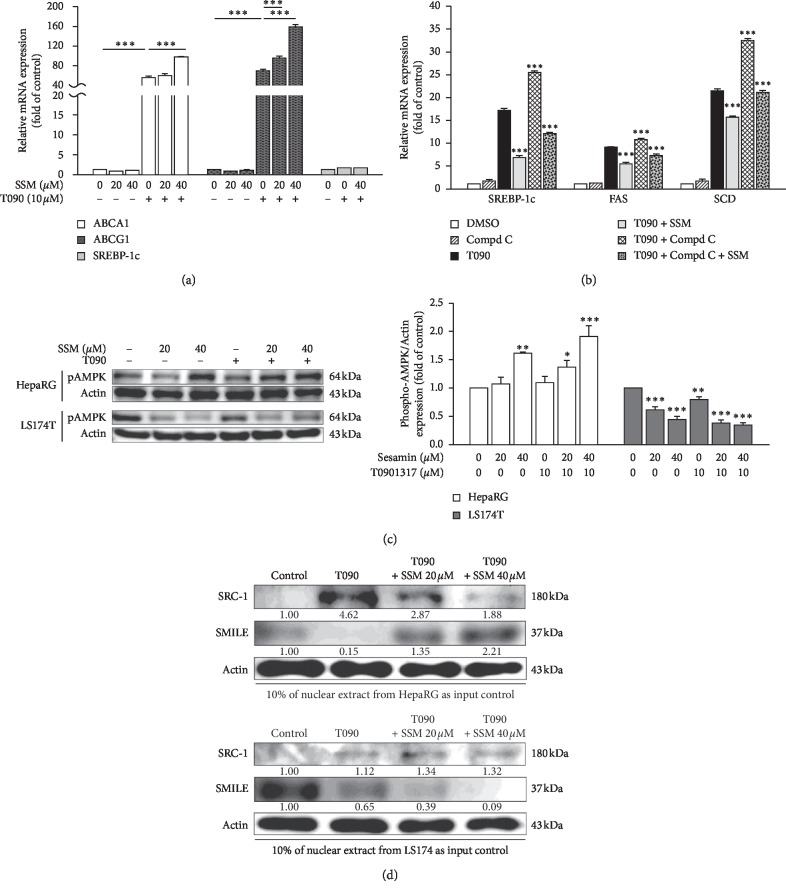
Sesamin (SSM) selectively induces reverse cholesterol transport- (RCT-) related genes in LS174T cells, affects AMPK signaling pathway, and differentially affects hepatocytes and intestinal cells. (a) and (b) LS174T cells were treated with SSM alone or in combination with T090 or compound C for 24 h. The expressions of RCT-related genes, *ABCA1* and *ABCG1*, *SREBP-1c*, *FAS*, and *SCD* were analyzed by real-time PCR. Data represent the mean ± SE; *n* = 3; ^*∗∗∗*^*p* < 0.001 compared with control or T090-treated groups as indicated. (c) Differentiated HepaRG cells or LS174T cells treated with SSM and T090, either individually or in combination for 24 h were harvested, and their protein levels of phospho-AMPK and *β*-actin (internal control) were analyzed via western blot. Quantitation of the indicated protein bands was corrected by *β*-actin expression. The representative blot shown was quantified with ImageJ software. Data represent the mean ± SE; *n* = 3; ^*∗*^*p* < 0.05; ^*∗∗*^*p* < 0.01; ^*∗∗∗*^*p* < 0.001 compared with the control group as indicated. (d) DNA binding affinity assay (DAPA) analysis at the SREBP-1c (upper) and ABCG1 (lower) response element from HepaRG and LS1714T cells. Five hundred micrograms of nuclear extracts, from SSM treatment in combination with 10 *μ*M T090, were used in DAPA experiments. A representative blot is presented, and the protein expression in untreated control cells was set to 1.

Our results so far revealed that SSM did not inhibit hepatic RCT-related gene expression, although it inhibited the expression of hepatic lipogenic genes. Then, we tested whether SSM may affect LXR*α*-related RCT gene expression in intestinal cells. To do so, we exposed LS174T cells to T090 and SSM treatments. As shown in Figure 4(a), *SREBP-1c* remained transcriptionally silent in these cells. However, the expressions of *ABCA1* and *ABCG1* in T090-treated cells were strongly enhanced by 56 and 69.4 folds compared with control cells. Interestingly, SSM did not inhibit LXR*α*-related activation of these RCT genes, but it further increased the expression of these genes by 97.4 and 158.8 folds (Figure 4(a)).

These results suggest that SSM might increase RCT in the intestinal cells through the activation of LXR*α*, and that may be beneficial for intestinal cells to improve cholesterol efflux. These different SSM modes of action in these two cell lines prompted us to investigate the potential roles of AMPK in the inhibition of LXR*α* activation by SSM. Compound C (6-(4-(2-piperidin-1-ylethoxy) phenyl)-3-pyridin-4-ylpyrazolo (1,5-a)pyrimidine) is the only available cell-permeable AMPK inhibitor [23]. Despite the existing controversy about its selectivity, compound C is still being widely used as an AMPK inhibitor. We applied it to investigate whether AMPK was involved in SSM-LXR*α* pathway via detection of the expression of LXR*α* target genes. As shown in Figure 4(b), mRNA levels of *SREBP-1c*, *FAS*, and *SCD* increased by 48%, 18%, and 51%, respectively, in cells exposed to both compound C and T090 as compared with T090-treated groups. However, SSM attenuated the upregulation of the genes. These observations suggest that AMPK is involved in this SSM-LXR*α* signaling pathway and partially regulates the expression of LXR*α* target genes in HepaRG cells. We further assessed AMPK activation in both cell lines in the presence of SSM alone or in combination with T090. The results showed that phospho-AMPK levels increased in HepaRG cells but decreased in LS174T cells in the presence of SSM (Figure 4(c)). Therefore, SSM exerts cell-type and gene-type-specific effects in HepaRG and LS174T cells and that other molecular pathways might be involved.

In order to investigate the involvement of two main coregulators (SRC-1 and SMILE) involved in SSM-LXR*α* signaling pathway, we used DAPA experiments to assess the recruitment of SRC-1 and SMILE to *SREBP-1c* and *ABCG1* probes which harbor LXR binding motif in the nuclear extract of HepaRG and LS174T under T090 and SSM treatment. We found that SRC-1 was bound to *SREBP-1c* LXRE region under T090 treatment; however, SSM treatment blocked this binding effect (Figure 4(d), upper panel). Contrastingly, recruitment of SMILE was decreased in T090-treated HepaRG cells, but it was rerecruited to the *SREBP-1c* LXRE binding motifs during SSM treatment. Interestingly, we found an opposite trend in LS174T cells, where SSM induced the recruitment of SRC-1 to the *ABCG1* LXRE binding motifs, but decreased that of corepressor SMILE binding to its binding sites (Figure 4(d), lower panel). These results suggest that SSM performed distinguishable LXR*α* signaling effects in hepatocytes and intestinal cells which may account for different outcomes of coregulators recruitment to the lipogenic and RCT gene promoters.

### 3.5. Sesamin Attenuated the Transactivation of LXR*α* by Valproate

**Figure 5:**
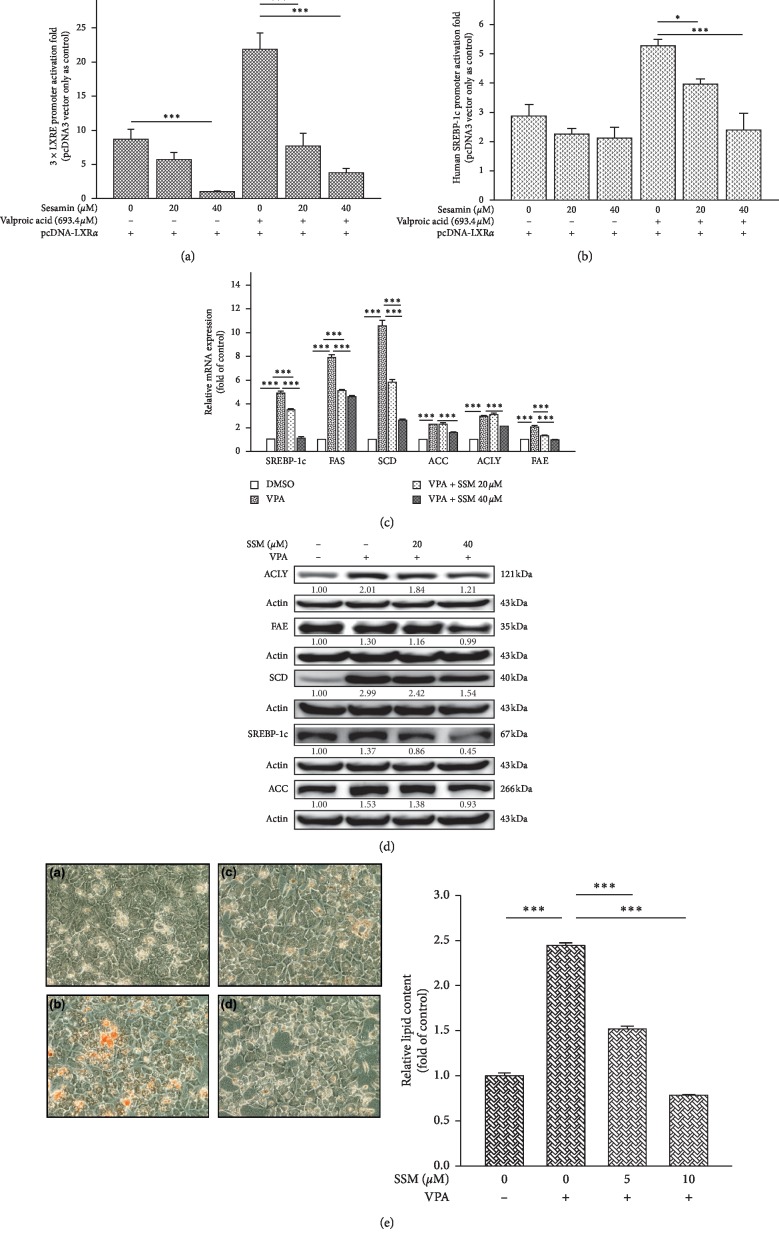
Sesamin (SSM) attenuates valproate- (VPA-) induced transient transcriptional assays of 3 × LXRE and SREBP-1c reporter activity, LXR*α* target genes mRNA and protein expressions, and lipid content. HepG2 cells were cotransfected with an LXR*α* expression plasmid and (a) 3 × LXRE-Luc and (b) SREBP-1c-Luc reporter genes and treated with 20 or 40 *μ*M SSM alone or in combination with 693.4 *μ*M VPA for 24 h. Data represent the mean ± SE; *n* = 4; ^*∗*^*p* < 0.05; ^*∗∗∗*^*p* < 0.001 compared with control or T090-treated groups as indicated. (c) Differentiated HepaRG cells treated with 20 or 40 *μ*M SSM alone or in combination with 693.4 *μ*M VPA for 24 h. Quantitative real-time PCR results of gene expression levels of *SREBP‐1c*, *FAS*, *SCD*, *ACC*, *ACLY*, and *FAE* are shown. *β-actin* was used as an internal control. Data represent the mean ± SE; *n* = 3; ^*∗∗∗*^*p* < 0.001 compared with control or VPA-treated groups as indicated. (d) Differentiated HepaRG cells treated with VPA and SSM alone or in combination for 24 h were harvested, and their protein levels of LXR*α* target genes and *β*-actin (internal control) were analyzed via western blot. Quantitation of the indicated protein bands was corrected by *β*-actin expression. The representative blot shown was quantified with ImageJ software. (e) Differentiated HepaRG cells were repeatedly exposed to (A) solvent, (B) 693.4 *μ*M VPA, (C) 693.4 *μ*M VPA + 5 *μ*M SSM, and (D) 693.4 *μ*M VPA + 10 *μ*M SSM for 14 days. Lipid accumulation was then assessed using Oil Red O staining, which allows the detection of triglycerides and cholesterol esters. HepaRG cells were observed and photographed under a phase‐contrast microscope (original magnification ×400). Oil red O dye was then extracted using isopropanol and quantified with a microplate reader at 510 nm. Values represent the mean ± SE; *n* = 3; ^*∗∗∗*^*p* < 0.001 compared with control or T090-treated groups as indicated.

In our previous work, we found that VPA may activate LXR*α* transactivation and lead to lipogenesis in hepatocyte-like cells [16]. Thus, we investigated whether the inhibitory effects of SSM may also attenuate VPA-mediated LXR*α* activation using 3 × LXRE, SREBP-1c promoter activity assays, and mRNA and protein expression measurements in differentiated HepaRG cells treated with SSM. We found that SSM decreased VPA-induced 3 × LXRE and SREBP-1c promoter activity in the presence of LXR*α* (Figures 5(a) and 5(b)). A set of LXR*α* target genes related to lipogenic processes, including *SREBP-1c*, *FAS*, *SCD*, *ACC*, *ACLY*, and *FAE*, had their expression induced by VPA, such upregulation was attenuated by SSM in a dose-dependent manner (Figure 5(c)). This was consistent with the findings on protein levels (Figure 5(d)). We confirmed that these alterations in protein and mRNA levels were associated with corresponding changes in lipid accumulation levels in these cells (Figure 5(e)).

### 3.6. Sesamin Attenuated the Transactivation of PXR by Rifampin

**Figure 6:**
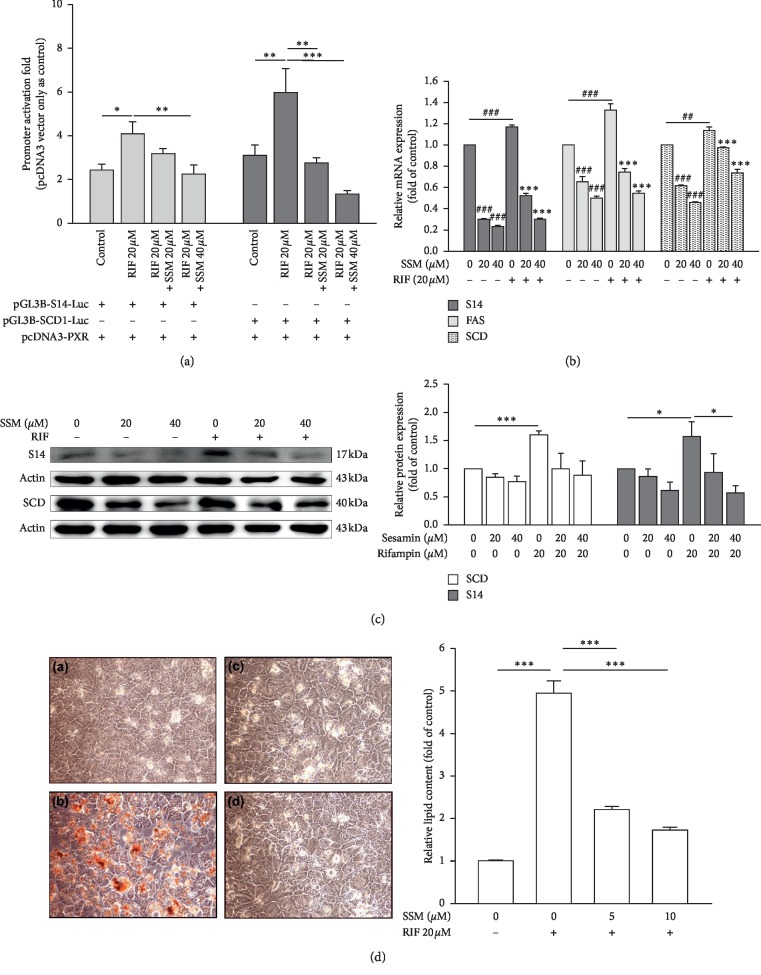
Sesamin (SSM) attenuates rifampin- (RIF-) induced transient transcriptional assays of S14 and SCD reporter activity, PXR target genes mRNA and protein expressions, and lipid contents. (a) HepG2 cells were cotransfected with a PXR expression plasmid and S14-Luc and SCD-Luc reporter genes and treated with 20 or 40 *μ*M SSM and in combination with 20 *μ*M RIF for 24 h. Data represent the mean ± SE; *n* = 4; ^*∗*^*p* < 0.05; ^*∗∗*^*p* < 0.01; ^*∗∗∗*^*p* < 0.001 compared with control or RIF-treated groups as indicated. (b) Differentiated HepaRG cells treated with 20 or 40 *μ*M SSM alone or in combination with 20 *μ*M RIF for 24 h. Quantitative real-time PCR (real-time PCR) results of gene expression levels of *S14*, *FAS*, and *SCD* are shown. *β-actin* was used as an internal control. Data represent the mean ± SE; *n* = 3; ^##/*∗∗*^*p* < 0.01; ^###/*∗∗∗*^*p* < 0.001 compared with control or RIF-treated groups, respectively, as indicated. (c) Differentiated HepaRG cells treated with RIF or SSM alone or in combination for 24 h were harvested, and their protein levels of PXR target genes and *β*-actin (internal control) were analyzed via western blot. Quantitation of the indicated protein bands was corrected by *β*-actin expression. The representative blot shown was quantified with ImageJ software. Data represent the mean ± SE; *n* = 3; ^*∗*^*p* < 0.05; ^*∗∗∗*^*p* < 0.001 compared with control or RIF-treated groups as indicated. (d) Differentiated HepaRG cells were repeatedly exposed to (A) solvent, (B) 20 *μ*M RIF, (C) 20 *μ*M RIF + 5 *μ*M SSM, and (D) 20 *μ*M RIF + 10 *μ*M SSM for 14 days. Lipid accumulation was then assessed using Oil Red O staining, which allows the detection of triglycerides and cholesterol esters. HepaRG cells were observed and photographed under a phase‐contrast microscope (original magnification ×400). Oil red O dye was then extracted using isopropanol and quantified with a microplate reader at 510 nm. Values represent the mean ± SE; *n* = 3; ^*∗∗∗*^*p* < 0.001 compared with control or RIF-treated groups as indicated.

Rifampin, a potent activator of PXR in humans, induces hepatic steatosis in patients with tuberculosis [3]. In our previous work, we found that SSM may inhibit the CYP3A4 expression through PXR-mediated pathway [24]. Thus, we analyzed the effects of SSM on PXR by assessing PXR-regulated lipogenic genes, including *S14*, *FAS*, and *SCD*. Using S14 and SCD reporter assays, we found that RIF induced their promoter activity, but these inductions were reduced by the cotreatment with SSM (Figure 6(a)). These inhibitions were corroborated by several experiments, including mRNA expression (Figure 6(b)), protein expression (Figure 6(c)), and lipid accumulation (Figure 6(d)) in HepaRG cells induced by RIF. The results showed that SSM changes the agonist-induced activity of both LXR*α* and PXR, suggesting that SSM may act as an antagonist of these NRs.

## 4. Discussion

In the present study, we provided several pieces of evidence indicating that sesamin is a naturally occurring LXR*α* and PXR antagonist. First, gene reporter assays showed that sesamin inhibited both LXR*α* and PXR transcription activities induced by either T090/VPA (for LXR*α*) and RIF (for PXR). Second, the mRNA and protein expression confirmed that sesamin attenuated the effects of LXR*α* and PXR agonists and reduced the expression of their downstream genes. Third, *in silico* modeling revealed that sesamin interacts with LXR*α* and thus reduces the induction caused by T090. We also found a novel pathway, in which sesamin activates AMPK in hepatocytes, but not in intestinal cells, reducing LXR*α* transactivation effects. Moreover, recruitment of SRC-1, but not SMILE, to the *SREBP-1c* promoter was observed in hepatocytes. On the other hand, we found increased recruitment of SMILE, but not SRC-1, binding to *ABCG1* promoter in intestinal cells. Thus, these mechanisms indicate that sesamin has distinct activities in these tissues and might have preferable effect of reducing hepatic steatosis and reducing absorption of cholesterol by intestine. This may be beneficial and underlie its atherogenic and antisteatotic actions.

Nuclear receptors are the second largest family of drug targets, and these receptors are involved in diverse physiologic functions linked to a wide range of diseases. Most importantly, the receptors are usually “drugable,” in the sense that it is feasible to identify small molecule modifiers to target these receptors for human therapy [25]. In the past 10 years, researchers have focused on the development of agonists to target LXR. As a lipid sensor, LXRs control the expression of genes involved in hepatic lipid metabolism. Treatment with the most promising LXR agonist, T0901317, causes severe hypertriglyceridemia and hepatic TGs accumulation [26]. On the other hand, recent reports have indicated that naturally occurring antagonists have the ability to reduce blood TG levels and might be beneficial for fatty liver improvement. It is suggested that using LXR*α* antagonists as a therapeutic agent is a promising treatment strategy. A comprehensive review from Komati [27] reported many LXR-related ligands are able to revert many pathological conditions, such as guttiferone, riccardin, naringenin, genistein, taurine, rhein, white button mushroom, morin, and so on. Recently, we also identified that oleanolic acid and ursolic acid are two newly identified LXR*α* antagonists [17, 28]. Herein, we show that sesamin also exerts LXR*α* antagonist effects. It may modulate lipid metabolism-related gene expression by suppressing the LXR*α*-mediated transcription of SREBP-1c. Additionally, sesamin exhibits a different activation mechanism on LXRs-RCT gene expression in intestinal cells by triggering the binding of coregulators on different promoter regions. Activation of AMPK is also involved in the sesamin-mediated LXR*α* inhibition pathway. Thus, sesamin potentially attenuates ligand-induced lipogenesis in hepatic cells and reduces absorption of lipids through intestinal route via different modes of action. Taken together, we can expect positive effects of sesamin in a hepatic steatosis model. A specific LXR ligand that does not induce lipid synthesis in the liver but retains the ability of RCT, such as sesamin, is of pharmacological and medical interest.

The worldwide spread of sedentary lifestyle and westernization of the diet have increased the prevalence of NAFLD in many countries. It is estimated that there are 20 million NAFLD patients nationwide [5]. The onset of fatty liver diseases may arise from: (1) increased lipid uptake; (2) elevated *de novo* lipogenesis; (3) impaired lipoprotein synthesis and secretion, (4) reduced catabolism of fatty acids by peroxisomal/mitochondrial *β*-oxidation, or (5) a combination of these [1]. People with fatty liver disease have a greater incidence of hyperglycemia (7% vs. 4%), hypertriglyceridemia (44% vs. 27%), hypercholesterolemia (54% vs. 44%), and decreased HDL-C (15% vs. 8%) compared with healthy counterparts [29]. They also have significantly greater serum levels of TGs and nonsignificantly greater levels of total cholesterol. In Taiwan, 25% to 37% of asymptomatic healthy subjects who undergo a health checkup have fatty liver. Serum TGs concentration above 130 mg/dL is a good predictor of fatty liver among Taiwanese people, and it is positively correlated with the severity of fatty liver [30]. Therefore, NAFLD is a detrimental condition that warrants the development of appropriate therapeutic interventions.

In addition to the induction of fatty liver disease, LXR activation is also involved in RCT, which is initiated by cholesterol removal from cells to HDL or to lipid-free apolipoproteins, such as apoAI or apoE. These are ultimately converted to bile acids in the liver excreted directly into the bile and decrease intestinal cholesterol absorption [31]. Repa et al. [8] demonstrated that T090 reduces intestinal dietary cholesterol absorption via increased intestinal ABCA1 expression. Thus, it helps to drive cholesterol from the enterocytes to the intestinal lumen, and the net cholesterol absorption is reduced. We found that expression of ABCA1/G1 increased markedly in LS174T intestinal cells after stimulation with T090, which agrees with a previous report by Hoang et al. [32]. Sesamin induced intestinal ABCA1/G1 mRNA levels especially in the presence of T090. The altered cholesterol transporter genes in the intestinal cells following sesamin stimulation may provide additional metabolic benefits during cholesterol homeostasis. This may also explain the reported antiatherosclerotic effects of sesamin.

The ligand-binding pocket of LXR*α* can adopt multiple conformations and accommodate an array of ligands with diverse shapes, structures, and volumes [33]. This characteristic makes LXR*α* a convenient target for the search for modulators to activate or inactivate it. We used a LXRE reporter gene assay and molecular docking to show that sesamin significantly inhibited T090-induced LXR*α* activation. Previous studies revealed that ARG305 of LXR*α* is an important residue that allows LXR*α* antagonists to form hydrogen bonds within the LBD of LXR*α*. The interaction with this specific residue, Arg305, may inhibit the binding of coactivators by rendering the H12 region [34]. Through virtual modeling, we showed that sesamin could dock into LXR*α* in a different manner compared with T090. This suggests that sesamin could act as a novel partial LXR*α*, providing insight for the design of novel LXR*α* modulators.

Ideally, clinically relevant LXR activators would be tissue- and gene-specific modulators with favorable antisteatotic and antiatherogenic properties without the less-favorable hepatic lipogenic effects. Additionally, LXR is known to elevate cholesterol levels in peripheral cells, the liver, and the intestine, which results in an overall net increase in cholesterol mobilization and catabolism, thus making LXR a pharmaceutical target for therapeutic intervention for the treatment of hypercholesterolemia and atherosclerosis. Indeed, several research groups identified agents that can reduce atherosclerosis without activating SREBP-1c or increasing hepatic lipogenesis, such as taurine, taurine, ethyl 2,4,6-trihydroxybenzoate, WAY-252623, and *N*,*N*-dimethyl-3*β*-hydroxy-cholenamide [32, 35, 36]. Indeed, sesamin enhances cholesterol efflux from RAW264.7 macrophages as reported by Liu et al. [37]. They found that sesamin can inhibit cholesterol accumulation via oxidized LDL (oxLDL) and stimulate cholesterol efflux from macrophages, RAW264.7 cells. The PPARγ-LXR*α*-ABCG1 pathway may be involved in this cholesterol efflux phenomenon. These findings explored the possibility that some of the antiatherosclerotic effects of LXR agonists may be independent of lipid metabolism in hepatocytes and may be attributable to direct actions on the vascular wall that activate RCT.

Recent research revealed that AMPK activation leads to inhibition of lipid synthesis and elevates fatty acid oxidation in hepatocytes, whereas it inhibits gluconeogenesis and stimulates glucose uptake and glycolysis [5]. Thus, it is suggested that AMPK downregulation in NAFLD causes excess accumulation of lipids in the patients' hepatocytes. In this study, we found that sesamin stimulates AMPK phosphorylation and thus decreases hepatic cells lipid accumulation. One of the mechanisms whereby AMPK may inhibit hepatic fatty acid synthesis is by suppressing SREBP-1c. Using a rat hepatoma model McA-RH7777, which mimics the intact liver through producing high levels of SREBP-1c, it was shown that activation of AMPK by AICAR and metformin suppresses SREBP-1c transcription by inhibiting endogenous LXR ligand production and SREBP-1c maturation processing [13]. The activation of AMPK activation attenuates the T090-mediated induction of endogenous SREBP-1c mRNA expression. These results indicate that AMPK directly inhibits ligand-induced LXR activation. We also observed that HepaRG cells treated with compound C and T090, the expressions of ABCA1/G1 were significantly upregulated compared with the cells treated with T090 only. In addition, metformin markedly reduces hepatic steatosis in humans, presumably acting through AMPK activation [38].

Previous studies showed that knockdown of SMILE gene expression increases the transactivation of several NRs, such as GR, CAR, NHF4*α*, LXR, FXR, and ERRγ [39]. The potential functional domains of SMILE that underlie its repressive function include the leucine zipper motif, the HCF-binding motif, and the LXXLL motifs. Lee and coworkers showed that SMILE reduced T090-induced hepatic lipid accumulation, TG levels, and lipogenic gene expression in high-fat diet-fed mice [11]. They concluded that SMILE is a potent modulator of hepatic lipogenesis by regulating LXR*α* through physical interaction, which inhibits the recruitment of LXR*α* to the SREBP-1c promoter by competing with its coactivator, SRC-1. Thus, SMILE acts as a corepressor of LXR*α*; this agrees with our findings on the repression of SREBP-1c and ABCG1 expression. We also demonstrated that SMILE-mediated LXR*α* target gene, SREBP-1c transcription, was affected by competition with SRC-1 on the *SREBP-1c* binding probe. Thus, the expression of lipogenic genes was inhibited by sesamin-induced AMPK phosphorylation and partly through recruitment of SMILE to the SREBP-1c promoter.

In the present study, we used human hepatoma HepaRG cells to demonstrate the occurrence of typical phenotypes of steatosis after drug treatment. This differentiated HepaRG cells which possess the unique capability to express most liver-specific functions at confluence, in contrast to HepG2 cells [40]. It is also a better cell model to analyze changes of gene expressions related to lipid homeostasis in order to better understand the molecular mechanisms of drug-induced steatosis. Drug-induced steatosis represents an important issue for the pharmaceutical industry during drug development; it may disrupt the development process or even cause withdrawal from the market. Valproate, which is commonly used to treat epilepsy, is a known steatosis inducer. It involves several mechanisms, including reduced fatty acid oxidation, increased *de novo* synthesis of fatty acids, and impaired secretion of lipids, causing cytosolic lipid accumulation in the liver [41]. Recently, we showed that VPA can transactive LXR*α* and thus cause lipogenic genes expression [18]. Herein, we demonstrated that sesamin was able to protect hepatocytes against VPA-induced hepatic lipogenic genes overexpression and lipid accumulation. Since the liver is the main metabolic engine of the body to metabolize drugs and other xenobiotics, it is susceptible to drug toxicity. Indeed, antiepileptic drugs have been reported to have unwanted adverse effects on the liver, as seen with VPA, phenytoin, and carbamazepine [42].

Pregnane X receptor (*NR1I2*) is an NR that regulates drug metabolism by sensing xenobiotics. It is abundantly expressed in the liver and intestine [20]. Indeed, cytochrome P450 3A4 (CYP3A4) is the main target of PXR; however, several genes involved in lipid homeostasis, such as SCD and S14, are also affected by PXR activation [19, 20]. Long-term stimulation of humanized PXR mice by rifampin caused obesity and hepatic steatosis via increased fatty acid uptake and synthesis and decreased *β*-oxidation [43]. In this study, we unveiled the protective effects of sesamin against rifampin-induced PXR activation of lipogenic genes, and thus it is a promising agent against drug-induced steatosis. Moreover, T090 is not a specific ligand towards LXR, and it also activates PXR [44]. In this study, we also showed the antagonistic effects of sesamin on PXR transactivation, suggesting that sesamin may also reverse the induction of PXR by rifampin and attenuate the development of steatosis clinically.

**Figure 7:**
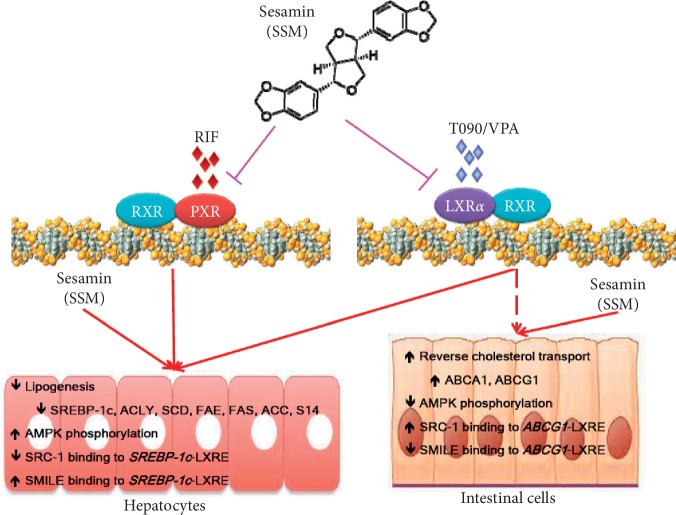
Schematic model of the LXR pathways in liver and intestinal cells and the action of sesamin on LXR*α* and PXR. Sesamin selectively reduced hepatic lipogenesis via the inhibition of SREBP-1c expression and its downstream target genes; however, genes involved in RCT of intestinal cells were preserved. It inhibited the hepatic lipogenesis partially via AMPK activation and increased SMILE recruitment to the SREBP-1c promoter. These effects were opposite in intestinal cells, where it did not recruit SMILE but competitively increased SRC-1 binding to the ABCG1 promoter region.

In summary, we demonstrated that sesamin is an antagonistic ligand of LXR*α*. Noteworthy, sesamin selectively reduces hepatic lipogenesis via the inhibition of SREBP-1c expression and its downstream target genes, but still preserving the genes which involved in RCT  of intestinal cells and hepatic cells while stimulation by LXR*α* agonist. Sesamin inhibits hepatic lipogenesis partially via AMPK activation and increase SMILE recruitment to the SREBP-1c promoter. These effects are oppositely observed in intestinal cells, where it does not recruit SMILE but competitively increased SRC-1 binding to the ABCG1 promoter (Figure 7). These interactions resulted in the net reduction of cellular neutral lipids in hepatocytes. In addition, the mechanism of coactivator/corepressor selectivity described here might be used to modulate the expression of other key regulatory proteins responsible for the pathogenesis of a diverse array of diseases. Our findings may provide better understanding of sesamin and associated chemicals and natural products in the treatment of liver disorders, especially NAFLD. It may have pharmaceutical and nutritional implications for the treatment of hypertriglyceridemia, fatty liver, and atherosclerosis in the future.

## Figures and Tables

**Table 1:** Sequences of PCR primers.

Gene	Species	Forward primer (5′–3′)	Reverse primer (5′–3′)
*hSREBP-1c*	Human	CGC TCC TCC ATC AAT GAC AA	TGC AGA AAG CGA ATG TAG TCG AT
*hFAS*	Human	ACA TCA TCG CTG GTG GTC TG	GGA GCG AGA AGT CAA CAC GA
*hSCD*	Human	CCG ACG TGG CTT TTT CTT CT	GCG TAC TCC CCT TCT CTT TGA C
*hACC*	Human	CTC TTG ACC CTG GCT GTG TAC TAG	TGA GTG CCG TGC TCT GGA T
*hACLY*	Human	GTG TGG ACG TGG GTG ATG TG	TTG ATG TCC TCA GGA TTC AGT TTC
*hFAE*	Human	TTC CGA GTC TCC CGG AAG T	ACA GCC CAT CAG CAT CTG AGT
*hABCA1*	Human	GAC ATC GTG GCG TTT TTG G	CGA GAT ATG GTC CGG ATT GC
*hABCG1*	Human	CGG TGC TCT CAT CCC TTT CA	GGT GTA CAG CCC GTC TTC CA
*hS14*	Human	TAT TTG CTC TGG CCC TTG CT	GGT CGC CAA GTA AGA GGG TG
*hβ-actin*	Human	CCT GGC ACC CAG CAC AAT	GCC GAT CCA CAC GGA GTA CT

## Data Availability

The data used to support the findings of this study are included within the article.

## Supplementary Materials

Supplementary Materials Figure S1: treatment protocol of T0901317 (T090), valproate (VPA), and rifampin (RIF) alone or in combination with sesamin (SSM) for lipid accumulation determination. Figure S2: cell viability of HepG2, HepaRG, and LS174T cells following exposure to sesamin (SSM) with or without T0901319 (T090) or rifampin (RIF). Figure S3: sesamin (SSM) inhibits the mRNA and protein expression of T0901317 (T090)-induced ABCG1 genes.
