# Supplementary material for: Sesamin, a Naturally Occurring Lignan, Inhibits Ligand-Induced Lipogenesis through Interaction with Liver X Receptor Alpha (LXRα) and Pregnane X Receptor (PXR)
Source: Evid Based Complement Alternat Med. 2019 Nov 25;2019:9401648. doi: 10.1155/2019/9401648 (PMC6959160; doi:10.1155/2019/9401648)
Supplement: Supplementary Materials — Figure S1: treatment protocol of T0901317 (T090), valproate (VPA), and rifampin (RIF) alone or in combination with sesamin (SSM) for lipid accumulation determination. Figure S2: cell viability of HepG2, HepaRG, and LS174T cells following exposure to sesamin (SSM) with or without T0901319 (T090) or rifampin (RIF). Figure S3: sesamin (SSM) inhibits the mRNA and protein expression of T0901317 (T090)-induced ABCG1 genes. [file 9401648.f1.pdf]

Evidence-Based Complementary and Alternative Medicine

Sesamin, a Natural Occurring Lignan, Inhibits Ligand-induced Lipogenesis through Interaction with Liver X Receptor Alpha (LXRα) and Pregnane X Receptor (PXR)

Tsai-Sung Tai, [1,§] Ni Tien, [2,3,§] Hsin-Yi Shen, [4] Fang-Yi Chu, [4] Charles C. N. Wang, [5] Chieh-Hsiang Lu, [1] Hui-I Yu, [1] Fang-Ping Kung, [1] Hsiang-Hsun Chuang, [1] Ying-Ray Lee, [6] Hsiao-Yun Chang, [7] and Yun-Ping Lim, [4,8,9]

[1] Department of Internal Medicine, Ditmanson Medical Foundation Chia-Yi Christian Hospital, Chiayi, 60080, Taiwan, R.O.C.

[2] Department of Laboratory Medicine, China Medical University Hospital, Taichung, 40458 Taiwan, R.O.C.

[3] Department of Medical Laboratory Science and Biotechnology, China Medical University, Taichung, 40458 Taiwan, R.O.C.

[4] Department of Pharmacy, College of Pharmacy, China Medical University, Taichung, 40458 Taiwan, R.O.C.

[5] Department of Bioinformatics and Medical Engineering, Asia University, Taichung, 41354 Taiwan, R.O.C.

[6] Department of Medical Research, Ditmanson Medical Foundation Chia-Yi Christian Hospital, Chiayi, 60080 Taiwan, R.O.C.

[7] Department of Biotechnology, Asia University, 41354 Taichung, Taiwan, R.O.C.

[8] Department of Internal Medicine, China Medical University Hospital, Taichung, 40458 Taiwan, R.O.C.

[9] Department of Medical Research, China Medical University Hospital, Taichung, 40458 Taiwan, R.O.C.

[§] These authors contributed equally to this work.

Correspondence should be addressed to Yun-Ping Lim; limyp@mail2000.com.tw

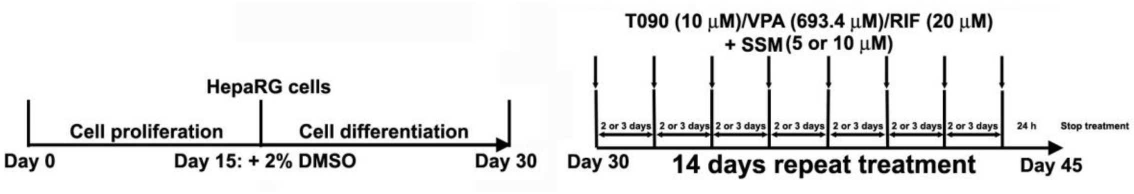

Figure S1. Treatment protocol of T0901317 (T090), valproate (VPA), and rifampin (RIF) alone or in combination with sesamin (SSM) for lipid accumulation determination. Human differentiated HepaRG cells were cultured for 2 weeks (proliferation phase) in an appropriate medium. The cells were subsequently transferred to the same medium supplemented with 2% (v/v) DMSO to induce cell differentiation. Schedules of repeated drug treatments were defined.

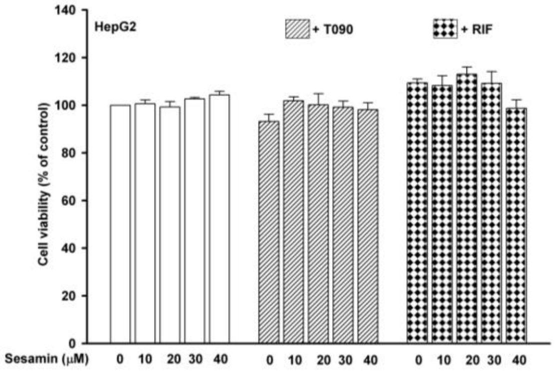

(**A**)

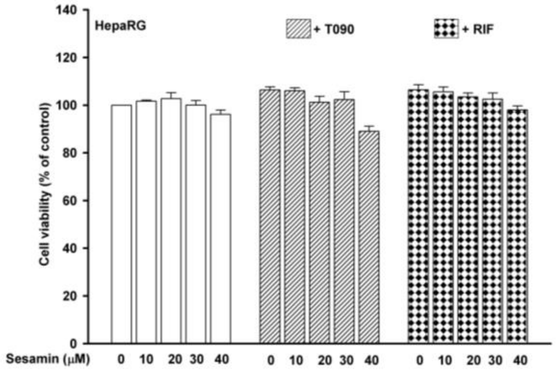

(**B**)

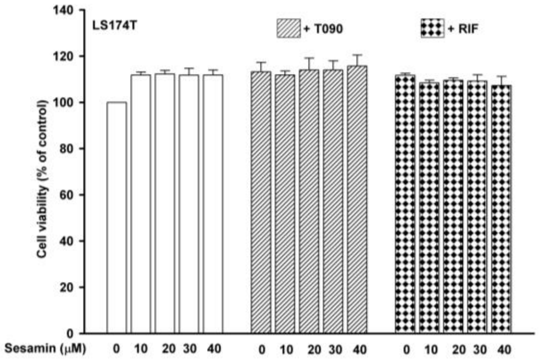

(**C**)

Figure S2. Cell viability of HepG2, HepaRG, and LS174T cells following exposure to sesamin (SSM) with or without T0901319 (T090) or rifampin (RIF). (**A**) HepG2, (**B**) HepaRG, and (**C**) LS174T cells were exposed to SSM (10–40 μM) alone or in combination with T090 (10 μM) or RIF (20 μM) for 24 h. Cell viability was monitored by cellular acid phosphatase activity using PNPP as a substrate. Data are shown as mean ± SE (n=3).

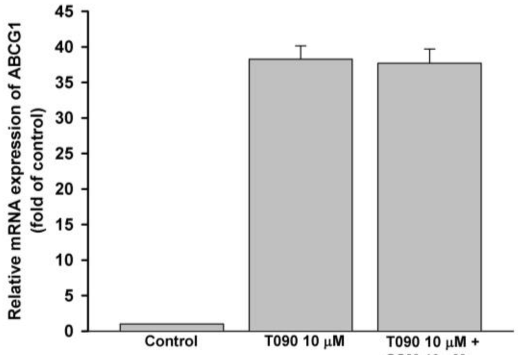

Figure S3. Sesamin (SSM) inhibits the mRNA and protein expression of T0901317 (T090)-induced ABCG1 genes. Differentiated HepaRG cells were treated with 40 μM SSM alone or in combination with 10 μM T090 for 24 h. Quantitative real-time PCR result of gene expression level of *ABCG1* is shown. *β-actin* was used as an internal control. Data represent the mean ± SE; n = 3; **, $p < 0.01$ compared with T090-treated group as indicated.
